# Supplementary material for: Chemical transport model simulations of organic aerosol in southern California: model evaluation and gasoline and diesel source contributions
Source: Atmos Chem Phys. Author manuscript; Available in PMC 2018 Aug 1. (PMC6070158; doi:10.5194/acp-17-4305-2017)
Supplement: Supp [file NIHMS982663-supplement-Supp.pdf]

Supplement of Atmos. Chem. Phys., 17, 4305–4318, 2017
http://www.atmos-chem-phys.net/17/4305/2017/
doi:10.5194/acp-17-4305-2017-supplement
© Author(s) 2017. CC Attribution 3.0 License.

Atmospheric Chemistry and Physics
Open Access

Supplement of

Chemical transport model simulations of organic aerosol in southern California: model evaluation and gasoline and diesel source contributions

Shantanu H. Jathar et al.

Correspondence to: Shantanu H. Jathar (shantanu.jathar@colostate.edu) and Allen L. Robinson (alr@andrew.cmu.edu)

The copyright of individual parts of the supplement might differ from the CC-BY 3.0 licence.

Table S1 Normalized mass emissions profiles for gasoline and diesel (columns 2 and 3) that map to CB05 model species in CMAQ for the VBS-IVOC simulation. Normalized emissions profiles for the Traditional and VBS simulations are provided in the supplementary material. SOA mass yields for CB05 model species for high and low NO_x conditions are listed in columns 3 through 10.

Species	Gasoline	Diesel	C* (high NO_x yields)				C* (low NO_x yields)			
			1	10	100	1000	1	10	100	1000
ALD2	0.0009	0.0020								
ALDX	0.0023	0.0131								
CH4	0.1816	0.0000								
ETH	0.0571	0.1911								
ETHA	0.0206	0.0173								
FORM	0.0030	0.0150	Do not produce SOA				Do not produce SOA			
IOLE	0.0094	0.0103								
OLE	0.0282	0.0596								
PAR	0.2670	0.3669								
UNR	0.0154	0.0429								
TOL	0.0574	0.0262	0.011	0.257	0.482	0.718	0.011	0.257	0.75	0.468
XYL	0.0844	0.0200	0.002	0.195	0.3	0.435	0.075	0.3	0.375	0.525
IVOC (gasoline)*	0.2723	0.0000	0.014	0.059	0.22	0.4	0.014	0.059	0.22	0.4
IVOC (diesel)*	0.0000	0.2330	0.044	0.071	0.41	0.3	0.044	0.071	0.41	0.3
ISOP	0.0003	0.0014	0	0.023	0.015	0	0.009	0.03	0.015	0
TERP	0.0000	0.0011	0.012	0.122	0.201	0.507	0.107	0.092	0.359	0.608
ALK5**	0.0988	0.0508	0	0.15	0	0	0	0.30	0	0
BENZ**	0.0345	0.0256	0.003	0.165	0.3	0.435	0.075	0.225	0.375	0.525

*IVOCs are assumed to have identical SOA mass yields for both low and high NO_x conditions

**In CB05, all alkanes are represented using the PAR species and benzene is not modeled as an explicit species in terms of gas-phase interactions. In the VBS-IVOC simulation, we consider emissions and SOA formation from the species ALK5 (representing long alkanes) and BENZ (benzene).

Table S2: Total emissions (tons) from May 4 to June 30, 2010 for NMOG, POA, BTEX (aromatics), ALK5 (long alkanes) and IVOCs (unspeciated SOA precursors) for all sources in the Los Angeles and Orange Counties for the three OA models: Traditional, VBS and VBS-IVOC.

Sources	***Traditional***				***VBS***				***VBS-IVOC***				
	NMOG	***POA***	***BTEX***	***IVOC****	***NMOG***	***POA***	***BTEX***	***IVOC****	***NMOG***	***POA***	***BTEX***	***IVOC****	***ALK5#***
Gasoline	11508	396	1986	0	12103	396	3360	595	11508	396	2513	2122	770
- On-road	6650	224	1172	0	6985	224	1831	336	6650	224	1395	1059	384
- Off-road	4859	172	814	0	5118	173	1529	259	4859	173	1118	1063	386
Diesel*	917	112	26	0	1085	112	67	168	917	112	60	102	22
- On-road	574	85	19	0	702	85	43	128	574	85	40	47	10
- Off-road	343	27	7	0	383	27	24	40	343	27	20	56	12
Other*	14476	839	1427	0	15748	848	2256	1273	14476	848	2254	1273	0
Total	26901	1346	3438	0	28937	1357	5684	2035	26901	1357	4827	3497	792
Biogenics	13531	0	0	0	13531	0	0	0	13531	0	0	0	0

*Does not include diesel engines used in railroad, marine and airport ground support equipment applications. These sources are linked to an emissions profile other than 8774.
**As described in the main manuscript, IVOCs are the same as the unspeciated SOA precursors.
#ALK5 as an SOA precursor is only included in the VBS-IVOC model

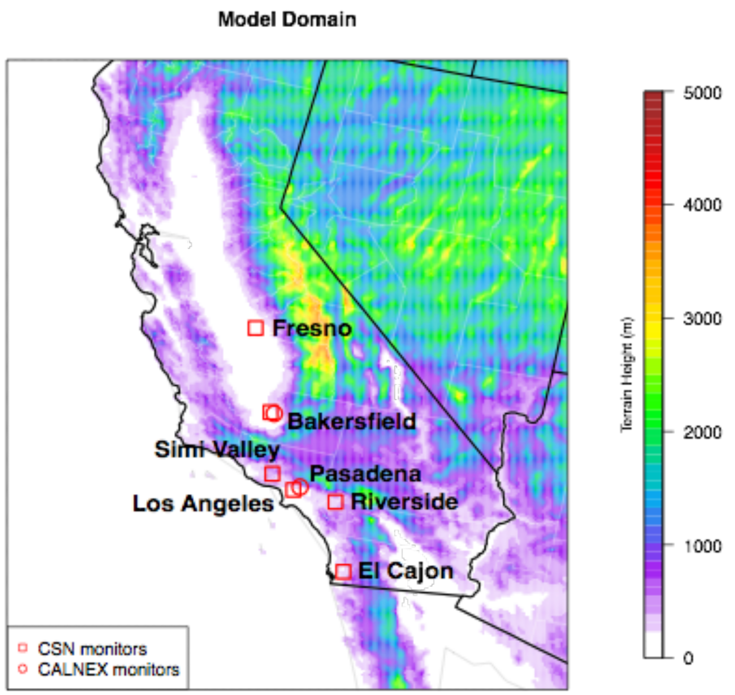

Figure S1: Model domain considered for the CMAQ simulations and locations of measurement sites.

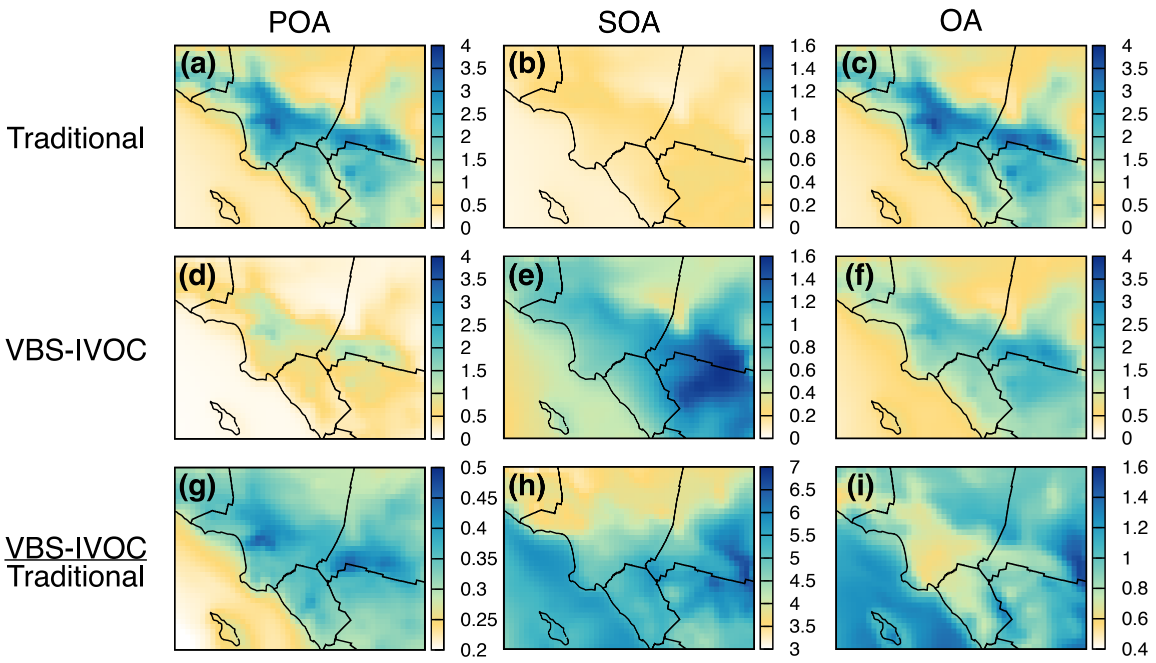

Figure S2: Comparison of campaign-averaged predictions of the Traditional model of Baker et al. (2015) and VBS-IVOC model. Left hand column (a, d, g) is primary organic aerosol (POA); middle column (b, e, h) is secondary organic aerosol (SOA); right hand column (c, f, i) is total organic aerosol (POA + SOA). Top row (a-c) shows predictions of the Traditional model; middle row (d-f) shows predictions of the VBS-IVOC model; bottom row (g-i) shows the ratio of the two model predictions.

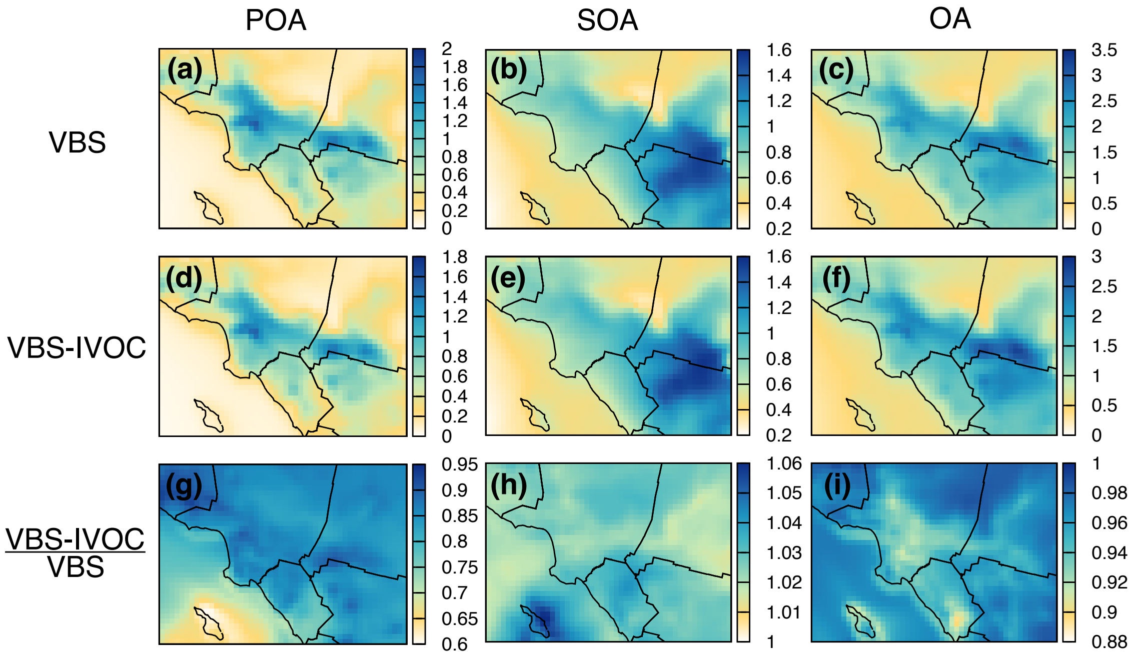

Figure S3: Comparison of campaign-averaged predictions of the VBS model of Woody et al. (2016) and VBS-IVOC model. Left hand column (a, d, g) is primary organic aerosol (POA); middle column (b, e, h) is secondary organic aerosol (SOA); right hand column (c, f, i) is total organic aerosol (POA + SOA). Top row (a-c) shows predictions of the VBS model; middle row (d-f) shows predictions of the VBS-IVOC model; bottom row (g-i) shows the ratio of the two model predictions.

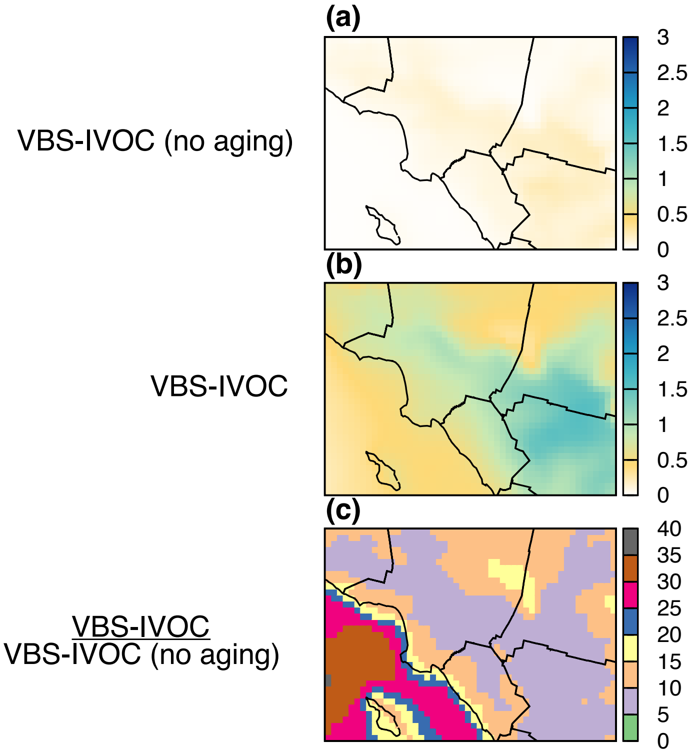

Figure S4: Comparison of campaign-averaged predictions of OA for the VBS-IVOC model (a) without aging reactions, (b) with aging reactions and (c) ratio of aging to no aging.
